# MiRNA-21 mediates the antiangiogenic activity of metformin through targeting PTEN and SMAD7 expression and PI3K/AKT pathway

**DOI:** 10.1038/srep43427

**Published:** 2017-02-23

**Authors:** Mao Luo, Xiaoyong Tan, Lin Mu, Yulin Luo, Rong Li, Xin Deng, Ni Chen, Meiping Ren, Yongjie Li, Liqun Wang, Jianbo Wu, Qin Wan

**Affiliations:** 1 Drug Discovery Reseach Center, Southwest Medical University, Luzhou, Sichuan, China; 2 Laboratory for Cardiovascular Pharmacology of department of Pharmacology, the School of Pharmacy, Southwest Medical University, Luzhou, Sichuan, China; 3 Affiliated TCM Hospital of Southwest Medical University, Luzhou, Sichuan, China; 4 Department of Internal Medicine, University of Missouri School of Medicine, Columbia, MO, USA; 5 Department of Endocrinology, the Affiliated Hospital of Southwest Medical University, Luzhou, Sichuan, China

## Abstract

Metformin, an anti-diabetic drug commonly used for type 2 diabetes therapy, is associated with anti-angiogenic effects in conditions beyond diabetes. miR-21 has been reported to be involved in the process of angiogenesis. However, the precise regulatory mechanisms by which the metformin-induced endothelial suppression and its effects on miR-21-dependent pathways are still unclear. Bioinformatic analysis and identification of miR-21 and its targets and their effects on metformin-induced antiangiogenic activity were assessed using luciferase assays, quantitative real-time PCR, western blots, scratch assays, CCK-8 assays and tubule formation assays. In this study, miR-21 was strikingly downregulated by metformin in a time- and dose-dependent manner. miR-21 directly targeted the 3′-UTR of PTEN and SMAD7, and negatively regulated their expression. Overexpression of miR-21 abrogated the metformin-mediated inhibition of endothelial cells proliferation, migration, tubule formation and the TGF-β-induced AKT, SMAD- and ERK-dependent phosphorylations, and conversely, down-regulation of miR-21 aggravated metformin’s action and revealed significant promotion effects. Our study broadens our understanding of the regulatory mechanism of miR-21 mediating metformin-induced anti-angiogenic effects, providing important implications regarding the design of novel miRNA-based therapeutic strategies against angiogenesis.

Metformin (N,N-dimethylbiguanide), an oral anti-hyperglycemic biguanide agent derived from *Galega officinalis*, has been used for decades in clinical therapy to treat metabolic disorders in type 2 diabetes (DM2) worldwide^1^,^2^. As an anti-diabetic drug, it is now well known that metformin potentiates insulin sensitivity and lowers blood pressure, glucose and triglycerides by inhibiting hepatic gluconeogenesis in DM2^3^. Recent retrospective studies have shown that metformin reduces the incidence and mortality of many common cancers, and diabetic patients treating with metformin showed a lower risk of cancer than those who treating with other antidiabetic drugs or no drugs^4^,^5^.

Recently, metformin has been proved to decrease some proangiogenic factors, thus, influencing angiogenesis, which is an essential step for organ growth, repair, tumor growth and metastasis, promotes the proliferation, migration and survival of endothelial cells by the angiogenic signaling^5^,^6^,^7^. The suppression of endothelial cell proliferation and migration contributes to the antiangiogenic activity, which has been revealed as a core component in clinically effective tumor therapy^5^,^8^. Furthermore, some studies have reported that metformin actions can activate adenosine monophosphate (AMP)-activated protein kinase (AMPK), and inhibit the mitochondrial activity and glycerophosphate dehydrogenase, in turn, leads to protein kinase signaling cascades that mediate the cellular energy charge and metabolism^1^,^2^,^5^,^6^,^7^. The activation of AMPK-independent pathways by metformin can inhibit the Akt activation and phosphorylation of Akt, interfering the Akt-signaling pathway and then regulating the cell proliferation, cell growth and cycle progression^1^,^9^,^10^.

The mechanism of metformin on endothelial function and angiogenesis are possibly regulated through interfering of Akt-dependent signaling pathway, and then prevent angiogenesisby reducing the pro-angiogenic, vascularisation, and levels of TGF-β1^1^,^11^,^12^. Although all these studies seemed to show the mechanisms of how metformin mediates the interfering of endothelial cell proliferation, migration and angiogenesis, more recently, newer and deeper insights into these mechanisms have been reported that metformin impacts endothelial function and angiogenesis possibly via its modulation of miRNA expression^2^,^13^,^14^.

MicroRNAs (miRNAs) are endogenous, 21~23 nucleotides and non-conding small RNAs, which act as key regulators of post-transcriptional gene expression, thereby regulating the multiple aspects of endothelial function and angiogenesis^13^,^15^. MiR-21, an oncomir by its oncogenic activity, has been well reported to be highly up-regulated in multiple tumors during the past several years^16^,^17^,^18^. However, more recently, the biological roles of miR-21 has been well investigated in cardiovascular biology and disease, and its levels are highly expressed in many cardiovascular cells, including vascular smooth muscle cells, and endothelial cells. Some basic and clinical researches have shown that miR-21 plays important roles in the angiogenesis inhibition, causing the inhibition of endothelial function, including cell proliferation and migration^16^,^19^,^20^,^21^.

Some studies have demonstrated that the biological functions of miR-21 can be changed by some drugs-induced in endothelial cells e.g. cardamonin^20^, rapamycin^19^, and isoflurane^21^, and then influence angiogenic processes. However, the mechanism of how miR-21 involves in metformin-induced changes of endothelial function is poorly understood. Thus, the aim of the present study is to investigate whether miR-21 has a potential effect on the metformin-induced suppression of the angiogenic activity on endothelial cells.

## Materials and Methods

### Cell culture and reagents

Human umbilical vein endothelial cells (HUVECs) were obtained from the American Type Culture Collection (Manassas, VA) and cultured in RPMI1640 (Gino Biomedical Technology), and supplemented with 10% (v/v) FBS, 1% (v/v) L-glutamine, 1 mM sodium pyruvate, 100 units ml^−1^ penicillin and 100 μg ml^−1^ streptomycin, and were kept in a humidified incubator under 5% (v/v) CO_2_, 95% (v/v) air atmosphere at 37 °C. After achieving 70–90% confluency, cells were serum starved (0.2% FBS) overnight. Cells were harvested by trypsin digestion, washed and resuspended in 0.2% FBS for use in cell proliferation and migration assay. Cells used were passaged between 3 and 7 in all experiments. Metformin (#D150959, 1,1-dimethylbiguanide hydrochloride, purity: >97% HPLC) was purchased from Sigma-Aldrich (St Louis, MO). miR-21 mimic, inhibitor and their negative control (NC) oligonucleotides were purchased from RiboBio (RiboBio Co. Ltd, Guangzhou). The Lipofectamine 2000 transfection reagent was purchased from Invitrogen (Carlsbad, CA). LY2157299, a potent inhibitor of TGF-βR1 signaling, was purchased from Selleck Chemicals (Houston, TX).

### Cell transfection

HUVECs (2 × 10^5^) were transfected with miR-21 mimic (40 nM), inhibitor (100 nM), and corresponding to respective NC in 6-well plates using Lipofectamine 2000 following the manufacturer’s instructions. All experiments were performed in triplicate. Cells were incubated with Lipofectamine–miRNA mixtures for 6 h before the medium was changed.

### Quantitative RT-PCR

Total RNA was extracted with Trizol reagent (Life Technologies, USA) using the standard method. cDNA synthesis was performed with 1 *μ*g of total RNA, using the M-MLV Reverse Transcription (Promega, USA) according to the manufacturer’s recommended conditions. The primer sequences of miR-21 specific stem-loop RT can be found in Supplementary Table S1. qRT-PCR was amplified using the miScript SYBR Green PCR Kit (TaKaRa, Dalian, China) and the ABI PRISM 7700 cycler (Applied Biosystems, Foster City, CA). Amplification reactions were performed as 95 °C for 10 s, and followed by 40 cycles at denaturing (95 °C for 5 s), 58 °C −60 °C for 10 s and 72 °C for 10 s, and the dissociation curve analysis of PCR products were determined in the final stage of 55 °C to 95 °C. RNU6B and 18 S rRNA were used as endogenous controls for miR-21 and its target mRNAs expression, respectively. All samples were performed in 3 biological replicates with 3 biological and technical replicates. All ratios-changes were calculated by using the 2^−△△CT^ mean ± SEM method^22^. The primers used in this study can be found in Supplementary Table S1.

### Cell Proliferation Assay

HUVEC proliferation was studied with Cell Counting kit 8 (CCK8, Beyotime) using in 96-well plates according to the manufacturer’s recommended protocol. Cells (3 × 10^3^) were added into wells, transfected with miR-21 mimic (40 nM), inhibitor (100 nM), and their respective NC, allowed to grow for 24 h at 37 °C. After medium was changed, the cells were treated with 20 mM metformin except for the control group, and then incubated for another 24 h at 37 °C. For each group, 6 duplicate wells were detected per experiment.

### Cell Migration Assay

HUVEC migration was studied using an *in vitro* scratch assay. Cells (1 × 10^6^/well) were seeded into 6-well plates, transfected with miR-21 mimic (40 nM), inhibitor (100 nM), and their respective NC for 24 h at 37 °C, followed by addition of 20 mM metformin and 0.2% FBS as a 24 h pre-treatment. A cell scratch spatula was performed using a sterile 200 μL pipette tip 3 times, respectively. Images of the scratches were taken using a digital camera system coupled to a microscope at the 0 h, 6 h, 12 h, and 24 h post-injury time points. The Image J software was used to determine the migration distance (μm) as the reduction of the width of the open area. At least 4 points in each of 3 random fields per well of 3 separate wound were examined.

### Tube Formation Assays

Formation of tubules by HUVECs was studied as described^6^, using 24-well plates coated with growth-factor-reduced Matrigel. About 1 × 10^5^/well HUVECs transfected with miR-21 mimic (40 nM), inhibitor (100 nM), and their respective NC were suspended and maintained in complete growth medium, and then were added into each coated well in the presence or absence of 20 mM metformin. Cells were incubated 24 h at 37 °C, washed, fixed, and viewed through an EVOS digital microscope (Electron Microscopy Sciences, Hatfield, PA, USA). Total tube length was measured in 5 fields (10×) using Image J software.

### Western blotting

Cell lysates were prepared as previously described^1^. After the bicinchoninic acid (BCA) assay, supernatants containing 25 μg of protein were subjected to 10% SDS/PAGE and transferred onto PVDF membrane (BioRad, CA, USA). After blocking, membranes were incubated with primary antibodies with rabbit or mouse IgG raised against PTEN, phospho-Smad2(Ser-465/467)/3 (Ser-423/425), SMAD2/3 (D7G7), phospho-Akt (Ser473), Akt (C67E7), phospho-ERK1/2 (Thr202/Tyr204), ERK1/2 (3A7), TGF-β, β-Actin, and the secondary antibody was horseradish peroxidase-(HRP-) conjugated goat anti-mouse IgG antibody raised against rabbit or mouse IgG. All antibodies were purchased from Cell Signaling (Danvers, MA, USA) except SMAD7 from Santa Cruz, CA, USA. Blots were developed by detection using ECL substrate (Pierce, Rockford, IL, USA). The Image J software was used to evaluate the band intensity.

### Luciferase reporter assay

Identification of miR-21 binding to the PTEN or SMAD7 3′-UTR was performed using the luciferase reporter assay as described previously^23^,^24^,^25^,^26^,^27^. The full-length of PTEN and SMAD7 mRNA 3′-UTR were RT-PCR-amplified and cloned into psi-CHECK2^TM^ vectors (Promega) using XhoIF, NotIR restriction sites. Mutagenesis of each of the flanks was performed using the Quikchange II Site-Directed Mutagenesis Kit (Stratagene). The wild-type or mutated fragments were then co-transfected a miR-21 mimic, inhibitor or control oligo into HEK 293 T cells using Lipofectamine 2000 (Invitrogen). After incubated for 48 h, the activity of FLuc/RLuc was then assessed using the Dual-Luciferase Reporter Assay System Kit (Promega, E1910). All experiments were performed at least three times. The relative activity of R-luc/f-Luc activity was calculated as the ratio of FLuc/RLuc in the miR-21 mimic or inhibitor transfected condition normalized to that in cells transfected with the control oligo condition.

### Statistical Analyses

Data were given as mean ± SEM. Analyses were performed in blinded fashion and conducted with IBM SPSS Statistics (version 20.0) software, using the unpaired Student’s t-test or one-way analysis of variance (ANOVA). *p < 0.05 were considered statistically significant.

## Results

### miR-21 expression was downregulated by metformin treatment

**Figure 1:**
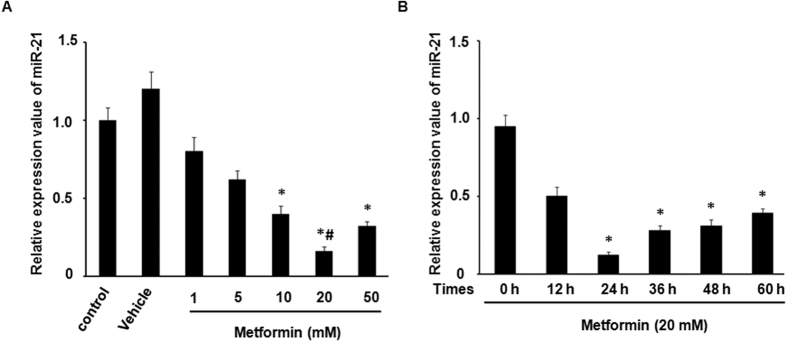
miR-21 was down-regulated by metformin treatment. (**A**) HUVECs were incubated with different concentrations (1 mM, 5 mM, 10 mM, 20 mM, and 50 mM) of metformin for 24 h. Control cells were untreated. Vehicle cells were treated with DMSO (0.05%). All data are presented as the mean ± SEM of triplicate independent experiments. ^*^p < 0.05 compared to control, ^#^*p* < 0.05 compared to 1, 5, 10, 50 mM metformin. (**B**) Time-dependent effects of metformin (20 mM) on relative miR-21 expression levels in HUVECs. All data are the mean ± SEM. ^*^p < 0.05 compared to 0 h (control).

To observe the effects of treatment with differential metformin concentration (1 mM, 5 mM, 10 mM, 20 mM, and 50 mM) and time length (0 h, 12 h, 24 h, 36 h, 48 h, and 60 h) on miR-21 expression, which were measured by the miRNA-specific stem-loop qRT-PCR. We found that miR-21 expression was downregulated significantly by metformin in a time- and does- dependent manner when compared with the control, and had expression nadirs at 24 h and 20 mM (Fig. 1A and B).

### Metformin-mediated inhibition of HUVECs proliferation, migration and tube formation through the intracellular level of miR-21

**Figure 2:**
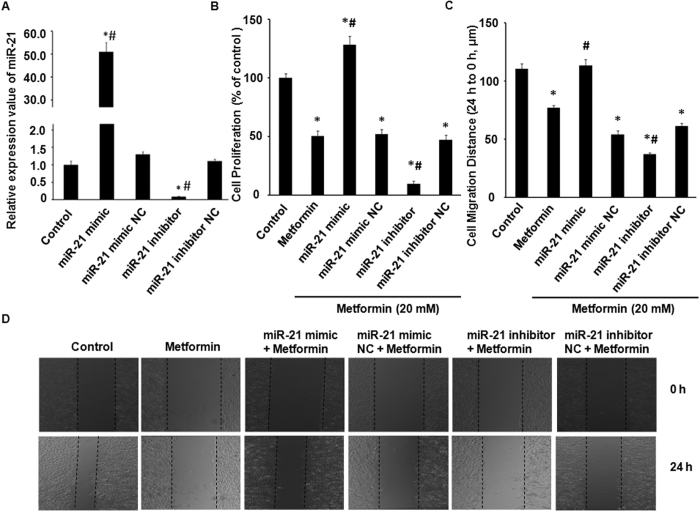
Metformin-mediated inhibition of HUVECs migration and proliferation through the intracellular level of miR-21. (**A**) Relative expression value of miR-21. (**B**) Cell counting kit-8 assay. HUVECs were first transfected with miR-21 mimic (40 nM). inhibitor (100 nM) or the NC, and then 5 × 10^3^ cells of each group were seeded in 96-well plates and treated with 20 mM metformin for 24 h. (**C** and **D**) *In vitro* wound healing assay. HUVECs were cultured in a 6-well plate, scratched, and transfected with miR-21 mimic/inhibitor and NC, and then treated with a medium containing 20 mM metformin. The migration assays were performed in media containing 0.2% FBS, which allows cell survival but not cell proliferation. All groups were photographed at the 0 h and 24 h, as quantified in (**C**). Scale bar in (**D**) = 1000 μm. ^*^p < 0.05 compared to control, ^#^*p* < 0.05 compared to metformin alone.

To determine whether transfection with miR-21 mimic or inhibitor can aggravate or attenuate the intracellular level of miR-21, we cultured HUVECs in wells transfected with miR-21 mimic (40 nM), inhibitor (100 nM), and corresponding to NC, respectively. After 24 hours, after which total cellular RNA was prepared, followed by cDNA synthesis, and analyzed by the stem-loop qRT-PCR. As shown in Fig. 2A, transfection with miR-21 mimic caused a significant increase in the expression of miR-21 in HUVECs, and conversely, transfection with miR-21 inhibitor reduced miR-21 levels significantly.

We examined the effects of miR-21 on the physiological responses of HUVECs to metformin treatment. The anti-proliferative effect of metformin was confirmed by testing the cell viability with a Cell Counting Kit-8 (CCK-8), which can reflect the proliferative ability of the HUVECs (Fig. 2B). Furthermore, when given in combination with metformin, the down-regulation of miR-21 by inhibitor caused a significantly stronger inhibition of HUVECs proliferation by 80.2% compared to metformin alone. Conversely, the overexpression of miR-21 by miR-21 mimic significantly abrogated metformin-mediated inhibition of HUVECs proliferation, and renewed by 154.9% compared to metformin alone (Fig. 2B).

The migration of HUVECs was measured with a scratch assay in this study. We found that metformin significantly inhibited HUVECs migration by 30.2% compared to controls (Fig. 2C,D), which was in accordance with some previous studies. To better investigate whether metformin’ inhibitory effects on HUVECs migration could be associated with the expression level of miR-21, when transfection with miR-21 mimic/inhibitor and respective NC given in combination with metformin treatment and 0.2% FBS (Fig. 2C,D), we found that inhibitor-induced miR-21 down-regulation caused a similar yet more effective inhibitory effect to metformin, and the migration inhibition was further down-regulated by another 21.6% compared to metformin alone, and otherwise, mimic-induced miR-21 over-expression can recover the metformin-inhibited HUVECs mobility, revealing a increase by another 16.4% compared to metformin alone.

More recently, metformin has been reported to inhibit HUVECs to form cord-like structures that mimic forming blood vessels^6^. To examine the effect of miR-21 on this process, HUVECs were first transfected with miR-21 mimic (40 nM), inhibitor (100 nM) and their respective NC, and then were cultured on Matrigel formed in the presence or absence of 20 mM metformin for 24 h. Metformin significantly inhibited the formation of capillary-like structures, as shown by the decrease of the tube length (Supplementary Figure S3A,B). Furthermore, down-regulation of miR-21 by inhibitor aggravated a significantly stronger inhibition of tubule formation compared to metformin alone, however, over-expression of miR-21 by mimic significantly abrogated the inhibition of HUVECs tubule formation compared to metformin alone (Supplementary Figure S3A,B). Both mimic- and inhibitor- negative control RNAs had no significant effects on tubule formation.

### PTEN and SMAD7 are direct targets of miR-21

We searched for putative miR-21 targets using Targetscan human software^28^ (http://www.targetscan.org/vert_71/), and the results were further confirmed using more efficient softwares, e.g., PicTar^29^ (http://pictar.mdc-berlin.de/), MicroRNA.org^30^ (http://www.microrna.org), and miRDB^31^ (http://mirdb.org/), suggesting that both PTEN and SMAD7 are potential targets of miR-21, which are in accordance with previous studies^23^,^24^,^25^,^26^,^27^.

**Figure 3:**
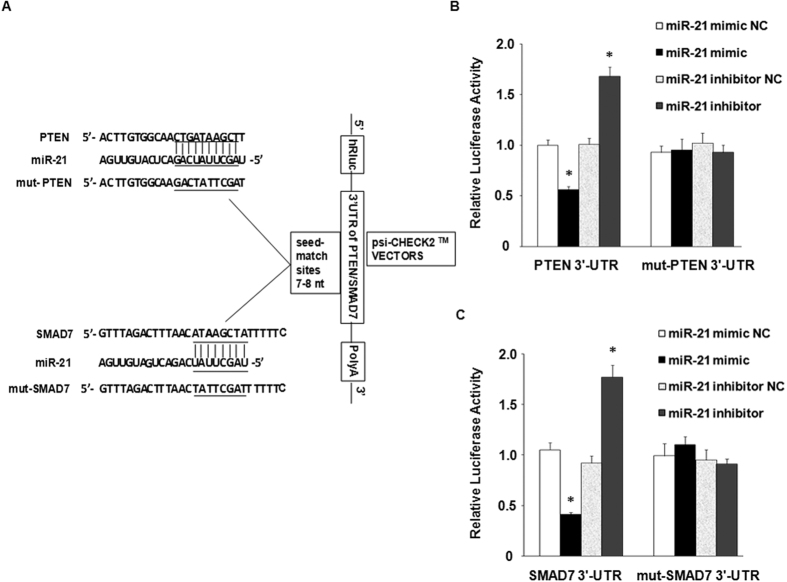
PTEN and SMAD7 are direct targets of miR-21. (**A**) Schematic representation of PTEN and SMAD7 3′-UTR luciferase reporter plasmids. The region nucleotides of PTEN and SMAD7 3′-UTR containing predicted target binding sites for miR-21 which was cloned at the 3′ end of luciferase reporter gene in psi-CHECK2^TM^ plasmids. The “seed sequences” and the point mutations in seed sequences were indicated by underbars. (**B** and **C**) A miR-21 mimic (30 nmol/L), miR-21 inhibitor (100 nM) or Negative Controls (NC 100 nM) were co-transfected with the psi-CHECK-2 wild-type or mutated PTEN and SMAD7 3′-UTR sequence vectors in HEK293 cells. The relative luciferase activity is reported. *p < 0.05, experimental groups *vs*. respective NC.

To further confirm that PTEN and SMAD7 are directly targeted by miR-21, we examined whether miR-21 directly bound to the 3′-UTR of PTEN and SMAD7 mRNAs by luciferase reporter gene assays. The 3′-UTR of PTEN and SMAD7 mRNAs containing the putative binding sites of miR-21, together with their corresponding mutated sequences, were cloned into the *Xho*I and *Not*I restriction sites of the psi-CHECK2^TM^ vectors, and co-transfected with miR-21 mimic, inhibitor or respective control oligos into HEK 293 T cells (Fig. 3A). We found that in the presence of the 3′-UTRs of PTEN and SMAD7, miR-21 mimic significantly suppressed the luciferase activity, conversely, miR-21 inhibitor significantly increased the luciferase activity (Fig. 3B and C). The effects of the miR-21 mimic and inhibitor were abrogated when mutated 3′-UTRs psi-CHECK2-constructs were used, suggesting that miR-21 could directly target PTEN and SMAD7 3′-UTRs.

### miR-21 regulates the expression of PTEN and SMAD7 levels in HUVECs

**Figure 4:**
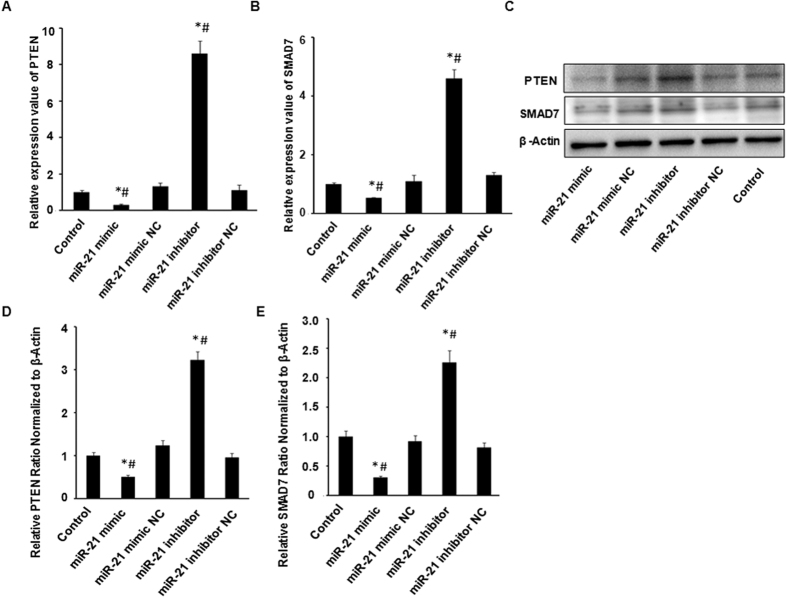
miR-21 regulates the expression of PTEN and SMAD7 levels in HUVECs. The expression levels of PTEN and SMAD7 mRNA (**A** and **B**) and protein (**C**,**D** and **E**) in HUVECs were significantly reduced by the over-expression of miR-21 in response to miR-21 mimic, and increased in response to miR-21 inhibitor, as determined using qRT-PCR and western blotting. All data are the mean ± SEM. *p < 0.05, experimental groups *vs*. control. ^#^*p* < 0.05, experimental groups *vs*. respective NC.

We then investigated whether there’re reciprocal changes of between miR-21 and PTEN/SMAD7 mRNA and protein levels in HUVECs. Expression levels of PTEN and SMAD7 were measured by qRT-PCR and western blotting in the previous cohort of HUVECs that were transfected with miR-21 mimic (40 nM), inhibitor (100 nM), and their respective NCs. The results showed that both PTEN and SMAD7 mRNA/protein levels were significantly reduced by over-expression of miR-21 in response to miR-21 mimic, conversely, down-regulation of miR-21 in response to miR-21 inhibitor significantly increased the expression levels of PTEN and SMAD7 (Fig. 4A,B,C,D and E). Taken together, these data indicated that miR-21 indeed negatively regulated PTEN/SMAD7 mRNA and their protein expressions by directly binding to their 3′-UTRs in HUVECs.

### Metformin reduces miR-21 expression through the TGF-β pathway

**Figure 5:**
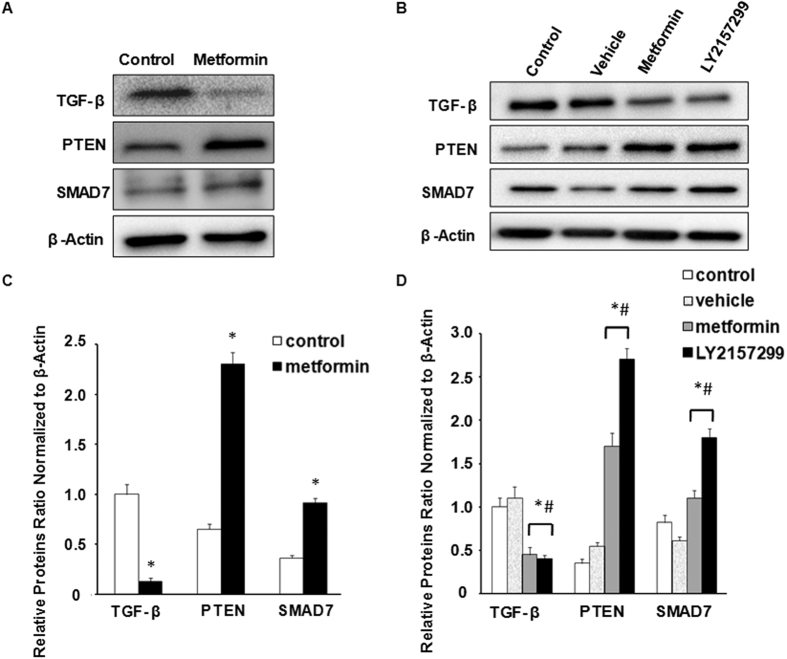
Metformin reduces miR-21 expression through the TGF-β pathway. (**A**,**B**,**C** and **D**) Western blotting analysis of the expression levels of TGF-β, PTEN and SMAD7 on HUVECs in response to metformin treatment and LY2157299 treatment. Control cells were untreated. Vehicle cells were treated with DMSO (0.05%). HUVECs were treated with 20 mM metformin and 10 μM LY2157299 for 24 h. β-Actin was used as a loading control. All graphs correspond to the blots above them and represent densitometric analyses of 3 independent experiments and are expressed as the mean ± SEM. *p < 0.05, experimental groups *vs*. control. ^#^*p* < 0.05, experimental groups *vs*. respective vehicle.

PTEN and SMAD7, as known targets of miR-21, have been shown to be the TGF-β pathway antagonists^11^,^25^,^32^,^33^. To evaluate whether metformin suppresses miR-21 expression through the repressors of TGF-β pathway, which induces an increase in PTEN and SMAD7 levels, we examined the expression levels of TGF-β, PTEN and SMAD7 on HUVECs in response to metformin treatment. In the presence of metformin, TGF-β protein expression was significantly reduced, and conversely, PTEN and SMAD7 protein expression levels were significantly increased, compared to the non- metformin treatment group, suggesting that metformin reveals a similar effect to miR-21 inhibitor on PTEN and SMAD7 protein expression levels (Fig. 5A and C).

Furthermore, when HUVECs were treated with LY2157299 (10 μM), a potent small-molecule antagonist of TGF-β receptor I kinase (TGF-βR1), high inhibitions of TGF-β expression were detected by metformin and LY2157299 treatments, and both PTEN and SMAD7 protein expression levels were significantly increased in response to metformin and LY2157299 (Fig. 5B and D). Treatment of LY2157299 (10 μM) also decreased the expression level of miR-21 in HUVECs (Supplementary Figure S1A). To determine whether blocking the TGF-β pathway would abrogate miR-21 mimic-induced metformin resistance in HUVECs, we studied the effect of the presence or absence of LY2157299 (10 μM) on the cell viability of HUVECs to miR-21mimic transfection and metformin treatment, CCK-8 assays showed that a strong dose of LY2157299 (10 μM) significantly inhibited the proliferative ability of the HUVECs, suggesting that LY2157299 treatment was sufficient to overcome miR-21 mimic-induced metformin resistance to the cell growth in HUVECs (Supplementary Figure S1B).

### Overexpression of miR-21 abrogates metformin-mediated inhibition of Akt, SMAD and ERK signaling pathways

Previous studies have shown that miR-21 acts as an important downstream regulatory factor of TGF-β^11^,^33^, which directly mediate the AKT-, SMAD- and ERK-dependent signaling pathways^34^, respectively. Therefore, we studied the differential time-dependent effects of metformin on protein phosphorylation pathways. We performed the experiments with HUVECs that were incubated with 20 mM metformin for 0 h (control), 15 min, 30 min, 1 h, 24 h, and 48 h, respectively, after which cell lysates were prepared to SDS-PAGE and western blotting analysis. Metformin (20 mM) caused significant decreases in p-Akt, and p-SMAD2/3 linker region after treatment from 15 min to 30 min in HUVECs, but no significant changes in the phosphorylation level of ERK1/2 (Supplementary Figure S2A and B).

**Figure 6:**
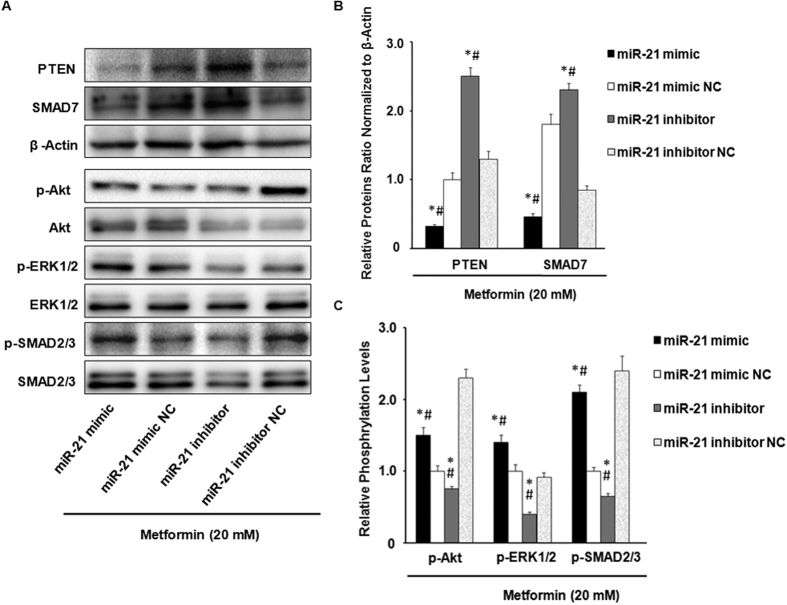
miR-21 regulates metformin-mediated inhibition of Akt -, SMAD- and ERK-dependent phosphorylation signaling pathways by targeting PTEN and SMAD7. (**A**,**B** and **C**) HUVECs were cultured in wells and transfected with miR-21 mimic (40 nM), inhibitor (100 nM), and their respective NC in the presence of 20 mM metformin. As shown, after which cell lysates were prepared and analyzed by western blotting using antibodies specific for PTEN and SMAD7 (**A** and **B**), and phosphorylated and total Akt, ERK1/2, and SMAD2/3 (**A** and **C**). All data are presented as the mean number per section ± SEM of 3 independent experiments. *p < 0.05, mimic groups *vs*. inhibitor. ^#^*p* < 0.05, experimental groups *vs*. respective NC.

To further evaluate whether overexpression of miR-21 by miR-21 mimic leading to down-regulation of PTEN and SMAD7 may abrogate the metformin-mediated inhibition of Akt -, SMAD- and ERK- phosphorylation signaling pathways, HUVECs were cultured in wells and transfected with miR-21 mimic (40 nM), inhibitor (100 nM), and their respective NC in the presence of 20 mM metformin. Under these conditions, the miR-21 mimic significantly inhibited PTEN and SMAD7 levels in HUVECs and efficiently increased the phosphorylation levels of Akt, SMAD and ERK, regardless of any effect of metformin treatment (Fig. 6A,B and D). In contrast, down-regulated of miR-21 (using the miR-21 inhibitor or metformin) significantly aggravated the upregulation of PTEN and SMAD7 levels and the metformin-mediated inhibition of protein phosphorylation pathways (Fig. 6A,B and D). These results suggested that overexpression of miR-21 could abolish the inhibitory effects of metformin-induced protein phosphorylations.

## Discussion

Endothelial cell functions such as proliferation and migration are the critical steps for angiogenesis, which is a complex process of generating new blood vessels, involving in a variety of human diseases including cancer growth and metastasis, diabetic complications and other vascular diseases^6^,^19^,^20^,^35^. To date, some studies have reported that inhibition of the endothelial cell proliferation and migration may enhance the antiangiogenic activity, which has been confirmed as a core component in clinically effective tumor therapy^6^,^36^. Several clinical studies have revealed that metformin can significantly decrease the risk of different types of cancer, and that this function was associated with its potent anti-proliferative and anti-angiogenic effects on HUVECs^5^,^6^,^7^. In addition, some miRNAs have been reported to be involved in the endothelial proliferation, migration and tube formation^19^,^37^,^38^. In this study, we tested the hypothesis that certain miRNA may be involved in mediating the process of metformin-induced inhibition of endothelial cell proliferation, migration and tube formation. We demonstrate for the first time that miR-21 is strikingly downregulated by metformin treatment in a time- and dose-dependent manner through the TGF-β pathway by targeting PTEN and SMAD7 in HUVECs. Overexpression of miR-21 with a miR-21 mimic abrogates the metformin-mediated inhibition of HUVECs proliferation, migration, tube formation and protein phosphorylations, suggesting that miR-21 can regulate the metformin-induced inhibitory effects of endothelial viability and mobility through regulating related cell signaling pathways.

Only few studies have reported the effects of metformin treatment on miRNAs expression in endothelial cells^2^,^13^. Here, we report that the expression of miR-21 is significantly downregulated by metformin treatment in a time- and dose-dependent manner in HUVECs, reaching the minimum levels at 24 h by a concentration of 20 mM of metformin treatment. Furthermore, it is clear from our findings that metformin causes potent inhibitory effects on HUVECs proliferation, migration and tube formation. This conclusion from the present study is in general accordance with previous studies^5^,^6^. However, the precise regulatory mechanisms by which the metformin-induced anti-proliferative and anti-angiogenic on HUVECs and its effects on miRNAs differential expression are still unclear, and further investigation is needed.

miR-21 has been reported to be an important regulator of angiogenesis, involving in mediating the proliferation and migration of vascular cells, including smooth muscle cells, and endothelial cells^16^,^18^,^19^,^20^. Direct studies have shown that downregulation of miR-21 expression significantly reduces the proliferation and migration of HUVECs, and conversely, miR-21 overexpression significantly enhances HUVECs proliferation and migration, indicating the importance of miR-21 on angiogenesis^19^,^20^. Recent studies have shown that metformin-mediated inhibition of proliferation and migration of other cell types is regulated by suppression of miR-21, which directly targets the 3′UTRs of PTEN, PDCD4, and Sprouty2 etc and negatively regulated their expression during the process^39^,^40^,^41^. Several studies have demonstrated that miR-21 can influence some clinical drugs-induced changes of the cell growth and migration ability in HUVECs^19^,^20^,^21^. In our present study, we demonstrate that down-regulation of miR-21 aggravates the metformin -mediated inhibition of HUVECs proliferation, migration and tube formation, while overexpression of miR-21 completely abrogates metformin’s action and reveals significant promotion effects on the proliferation, migration and tube formation of HUVECs, suggesting that miR-21 plays a key role in anti-proliferative, anti-migratory and antiangiogenic effects on metformin treated HUVECs.

To explore the putative mechanism for the involvement of miR-21 in the metformin-induced inhibition of HUVECs proliferation, migration and tube formation, we focus on the identification of target genes potentially regulated by miR-21. Our present study indicates that miR-21 directly targets the 3′-UTRs of PTEN and SMAD7, and negatively regulates their mRNA and protein expression levels in HUVECs. Our results are further supported by previous studies, which have demonstrated that both PTEN and SMAD7 are directly targeted by miR-21 in different cell types^24^,^25^,^26^,^27^,^33^.

Previous studies have shown that miR-21 acts as an important repressor of AKT-, and SMAD-dependent the TGF-β pathway by targeting PTEN and SMAD7, while the activation of miR-21 expression can be mediated by a TGF-β activity^11^,^26^,^33^. Metformin treatment has been recently found to cause an activation of AMPK-signaling, but also suppress the TGF-β-signaling, which appears to involve in inducing pro-sclerotic AKT- and SMAD3-mediated signaling pathways^1^,^3^,^5^,^6^,^7^,^9^. Our present study indicates that inhibition of the TGF-β pathway by metformin suppresses the expression of miR-21. Furthermore, blocking the TGF-β pathway is sufficient to overcome miR-21 overexpression-induced metformin resistance to the cell growth in HUVECs, suggesting that metformin reduces miR-21 expression through the TGF-β- signaling pathway. Results from the present study further demonstrate that metformin signifcantly suppresses the TGF-β-induced AKT-, SMAD- and ERK-dependent phosphorylation signaling pathways, but miR-21 overexpression inhibiting PTEN and SMAD7 levels can abrogate the inhibitory effects of metformin-induced protein phosphorylations, and conversely, down-regulated of miR-21 increasing PTEN and SMAD7 levels aggravates the metformin-mediated inhibition of protein phosphorylation pathways.

In conclusion, we have reported for the first time, that miR-21 regulates the metformin-induced suppression of endothelial proliferation, migration and tube formation through the TGF-β-induced AKT-, SMAD- and ERK-dependent phosphorylation signaling pathways by targeting PTEN and SMAD7. The overexpression of miR-21 abrogates the inhibitory effects of metformin treatment on the proliferation, migration, tube formation and protein-signaling phosphorylations of HUVECs, and down-regulated of miR-21 aggravates the process of metformin-induced inhibitory effects on HUVECs. Overall, our results broaden our understanding of the regulatory mechanism of miR-21 involves in mediating metformin-induced antiangiogenesis effects, leading to providing novel prognostic and predictive factors for cancer, diabetic complications and other vascular diseases, and the design of novel miRNA-based therapeutic strategies against angiogenesis. These results also support proceeding to additional preclinical studies using drug combinations for metformin, miR-21 inhibitor or mimic as therapeutic strategies to suppress or promote angiogenesis *in vivo*.

## Additional Information

**How to cite this article:** Luo, M. *et al*. MiRNA-21 mediates the antiangiogenic activity of metformin through targeting PTEN and SMAD7 expression and PI3K/AKT pathway. *Sci. Rep.* **7**, 43427; doi: 10.1038/srep43427 (2017).

**Publisher's note:** Springer Nature remains neutral with regard to jurisdictional claims in published maps and institutional affiliations.

## Supplementary Material

Supplementary Information
