# Supplementary material for: MiRNA-21 mediates the antiangiogenic activity of metformin through targeting PTEN and SMAD7 expression and PI3K/AKT pathway
Source: Sci Rep. 2017 Feb 23;7:43427. doi: 10.1038/srep43427 (PMC5322530; doi:10.1038/srep43427)
Supplement: Supplementary Information [file srep43427-s1.pdf]

Supplementary Information for

MiRNA-21 mediates the antiangiogenic activity of through targeting PTEN and SMAD7 expression and PI3K/AKT pathway

Mao Luo[1, 2§], Xiaoyong Tan[1, 2§], Lin Mu[3], Yulin Luo[1, 2], Rong Li[1, 2], Xin Deng[1, 2], Ni Chen[1, 2], Meiping Ren[1, 2], Yongjie Li[1, 2], Liqun Wang[1, 2], Jianbo Wu[1, 2, 5*], Qin Wan[4*]

[1] Drug Discovery Reseach Center, Southwest Medical University, Luzhou, Sichuan, China.

[2] Laboratory for Cardiovascular Pharmacology of department of Pharmacology, the School of Pharmacy, Southwest Medical University, Luzhou, Sichuan, China.

[3] Affiliated TCM Hospital of Southwest Medical University, Luzhou, Sichuan, China.

[4] Department of Endocrinology, the Affiliated Hospital of Southwest Medical University, Luzhou, Sichuan, China.

[5] Department of Internal Medicine, University of Missouri School of Medicine, Columbia, MO.

[§] These authors contributed equally to this work.

[*] Correspondence: Qin Wan, Department of Endocrinology, the Affiliated Hospital of Southwest Medical University, Luzhou 646000, China. Email: wanqin3@163.com.

Supporting Information

Supplementary Figure S1. Blocking the TGF-β pathway by LY2157299 treatment abrogates miR-21 mimic-induced metformin resistance. (A) Blocking the TGF-β pathway by LY2157299 treatment reduces the expression level of miR-21, as determined by qRT-PCR normalized to U6. $^{*}p<0.05$ compared to control, $^{\#}p<0.05$

compared to vehicle. (B) LY2157299 treatment abrogates miR-21 mimic-induced metformin resistance of the cell viability of HUVECs by a CCK-8 assay. All data are presented as the mean number per section ± SEM. **p* <0.05, experimental groups *vs*. control or respective NC, $^{\#}p<0.05$, miR-21 mimic *vs*. metformin alone, $^{\S}p<0.05$, miR-21 mimic + metformin *vs*. metformin alone, $^{\P}p<0.05$, miR-21 mimic + metformin + LY2157299 *vs*. miR-21 mimic + metformin, $^{**}p<0.05$, miR-21 mimic + metformin + LY2157299 *vs*. miR-21 mimic alone. Groups other than those denoted by an asterisk did not differ significantly.

Supplementary Figure S2. The differential time-dependent effects of metformin on phosphorylation pathways. (A and B) HUVECs were incubated with 20 mM metformin for 0 h (control), 15 min, 30 min, 1 h, 24 h, and 48 h, respectively, after which cell lysates were prepared to SDS-PAGE and western blotting analysis to see the expression of p-Akt, p-ERK1/2, and p-SMAD2/3. All data are presented as the mean number per section ± SEM. **p* <0.05, experimental groups *vs*. control.

Supplementary Figure S3. Metformin-mediated inhibition of HUVEC tube formation through the intracellular level of miR-21. (A and B) In vitro tube formation assay. HUVECs (1×10^5/well) were first transfected with miR-21 mimic (40 nM), inhibitor (100 nM) and their respective NC, and then, representative images of HUVECs grown on matrigel in the presence or absence of 20 mM metformin were shown as indicated for 24 h. Quantitative assessment of triplicate tubule formation experiments was performed. Total tube length was measured in 5 fields (10×) using ImageJ software. All data are presented as the mean number per section ± SEM of 3 independent experiments, presented as a percent of the control value (first bar). Scale bar in=500 μm. $^{*}p<0.05$ compared to control, $^{\#}p<0.05$ compared to metformin alone.

Supplementary Table S1. Sequences of primers used in the study.

Supplementary Figure S1. Blocking the TGF-β pathway by LY2157299 treatment abrogates miR-21 mimic-induced metformin resistance.

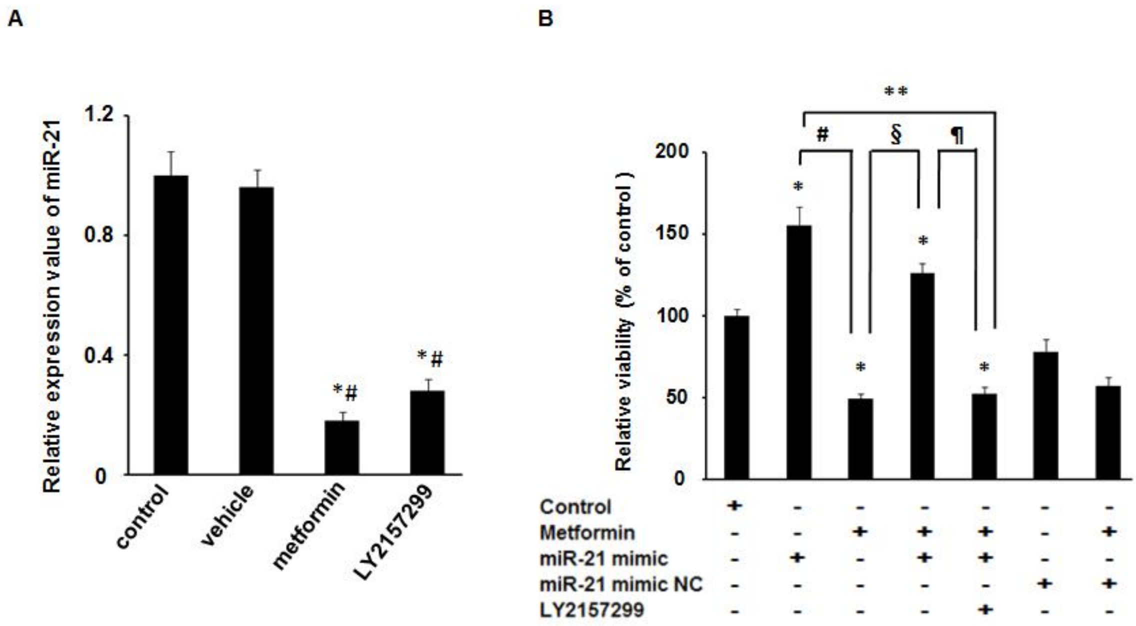

Supplementary Figure S2. The differential time-dependent effects of metformin on phosphorylation pathways.

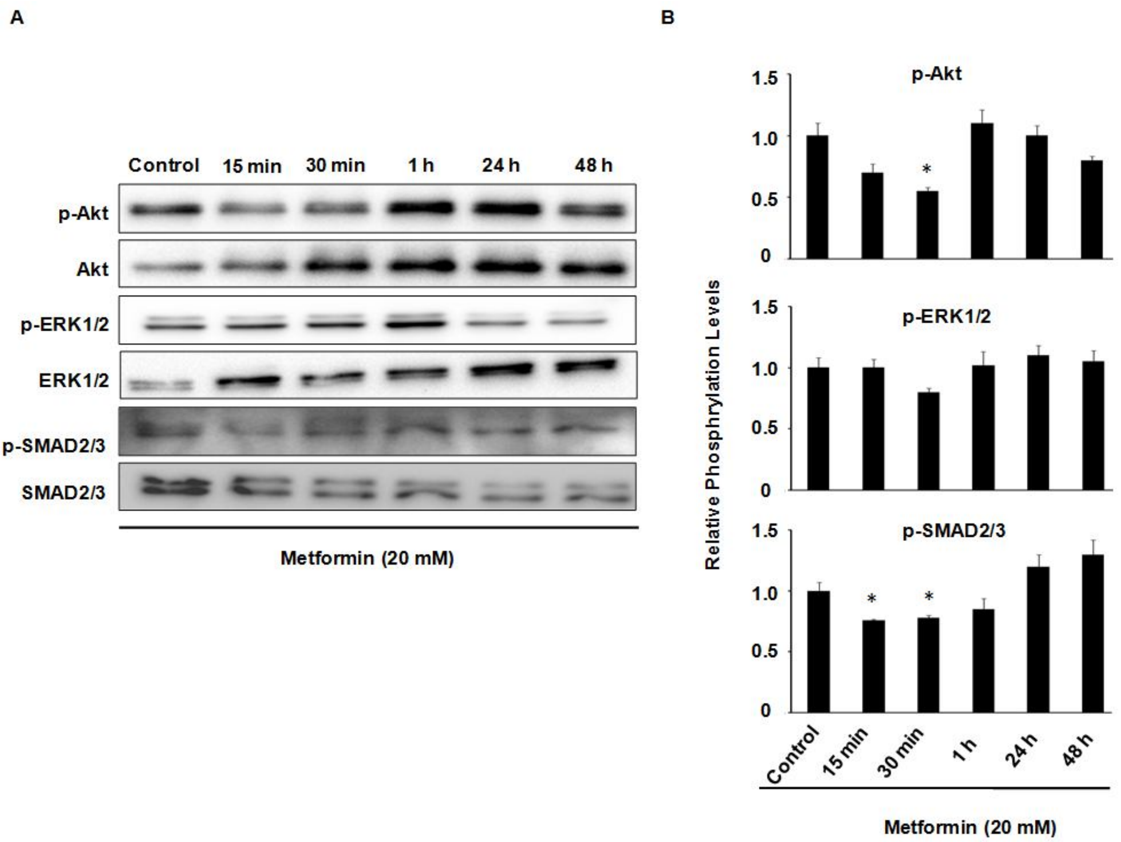

Supplementary Figure S3. Metformin-mediated inhibition of HUVEC tube formation through the intracellular level of miR-21.

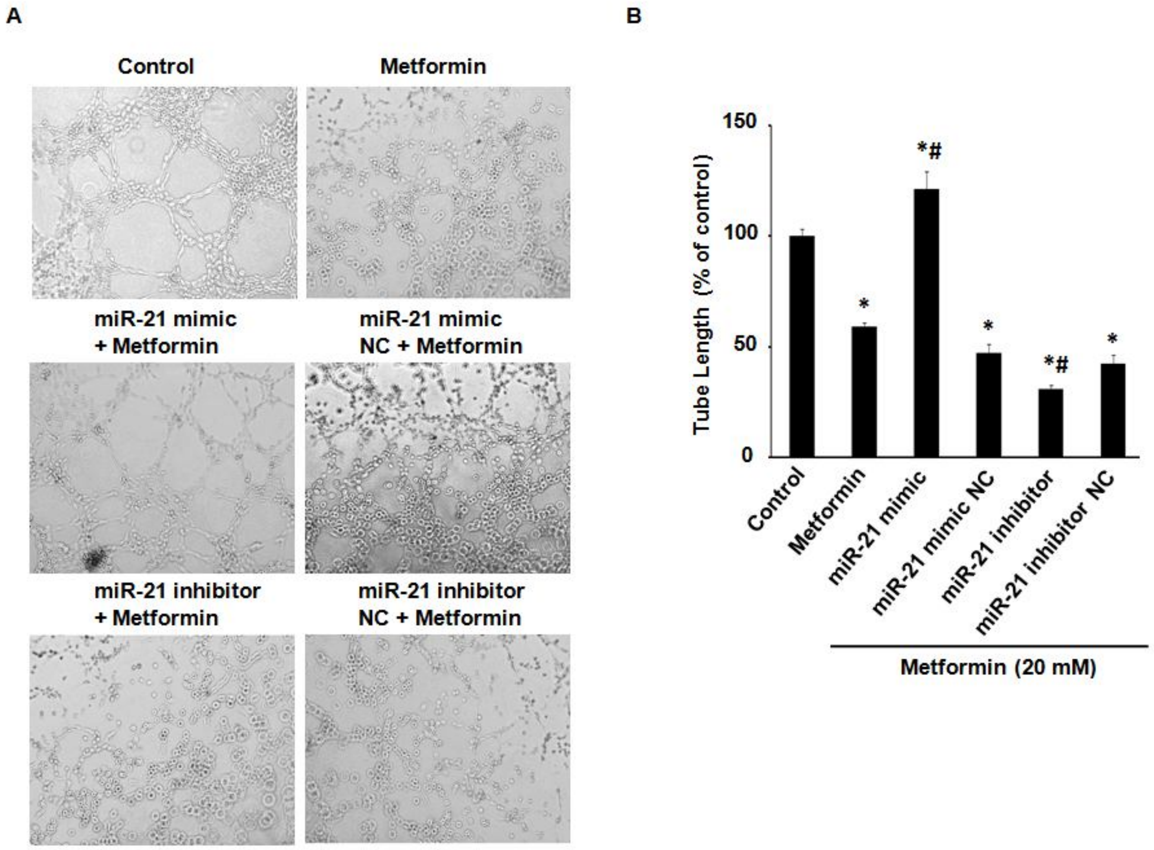

Supplementary Table S1. Sequences of primers used in the study.

	Primer	Sequences
Stem-loop Q-RT-PCR for miR-21	Stem-loop RT primer of miR-21	5'-CTCAACTGGTGTCGTGGAGTCGGCAATTCAGTTGA GTCAACATC-3'
	Sense primer of miR-21	5'-ACACTCCAGCTGGGTAGCTTATCAGACTGATG-3'
	Antisense primer of miR-21	5'- CTCAACTGGTGTCGTGGA -3'
	Sense primer of reference gene U6	5'-CTCGCTTCGGCAGCACA-3'
	Antisense primer of reference gene U6	5'-AACGCTTCACGAATTTGCGT-3'
Real-time PCR for human PTEN and SMAD7	Sense primer of PTEN	5'- TTGTGGTCTGCCAGCTAAA -3'
	Antisense primer of PTEN	5'- CGCTCTATACTGCAAATGCT -3'
	Sense primer of SMAD7	5'- GCTCCCATCCTGTGTGTTAA -3'
	Antisense primer of SMAD7	5'- TAGGTGTCAGCCTAGGATGGT -3'
	Sense primer of reference gene18S rRNA	5'-CCTGGATACCGCAGCTAGGA-3'
	Antisense primer of reference gene 18S rRNA	5'-GCGGCGCAATACGAATGCCCC-3'
